# Application of the Monaco-serial biological function for cardiac dose constraints in DIBH-IMRT treatment planning for left-sided breast cancer

**DOI:** 10.3389/fonc.2025.1610980

**Published:** 2025-06-09

**Authors:** Haili Hu, Zhou Jueyi, Jiang Hao, Jianjun Lai

**Affiliations:** ^1^ Zhejiang Chinese Medical University, Hangzhou, Zhejiang, China; ^2^ Zhejiang Hospital, Hangzhou, Zhejiang, China; ^3^ Lishui People’s Hospital, Lishui, Zhejiang, China; ^4^ Hangzhou Cancer Hospital, Hangzhou, Zhejiang, China; ^5^ Affiliated Hangzhou First People’s Hospital, School of Medicine, Westlake University, Hangzhou, China

**Keywords:** breast cancer, radiotherapy planning, biological optimization, monte carlo, equivalent biological dose

## Abstract

**Background:**

The Serial function in the Monaco treatment planning system is essential for cardiac dose optimization in left breast cancer radiotherapy; however its optimal K-value for deep-inspiration breath-hold intensity-modulated radiotherapy (DIBH-IMRT) has not been established. This study aims to determine the evidence-based K-value configuration for clinical implementation.

**Methods:**

41 left breast cancer patients undergoing DIBH-IMRT were retrospectively analyzed. Plans were stratified by Monaco-Serial K-values: Group A (K=1), B (2≤K ≤ 4), and C (K>4). Dosimetric parameters (heart, LAD, Lung-L) and dose-volume reduction rates (Groups B/C vs A) were compared. Correlations between K-values and DIBH-induced anatomical changes (Lung-L volume increment rate, Lung-L/Heart volume ratio, and Heart-Breast Distance increment) were assessed

**Results:**

All plans satisfied target coverage. Group B achieved optimal cardiac protection: mean heart dose (273.9 ± 91.0 cGy), max heart dose (2676.2 ± 1380.7 cGy), and LAD doses (mean: 411.3 cGy; max: 1483.3 ± 736.3 cGy) significantly decreased versus Group A. Lung-L V500cGy in Group B increased marginally but within clinical tolerance. Correlation analysis confirmed that Group B achieved balanced control of mean/maximum heart doses, aligning with the expected effects of anatomical variations induced by the DIBH technique.

**Conclusions:**

Adjusting Monaco-Serial K-value to 2≤K ≤ 4 provides optimal dose constraints for the heart and substructures while ensuring target coverage, making it the optimal parameter setting for left breast cancer DIBH-IMRT.

## 1 Introduction

Breast cancer is the most common malignancy among women worldwide, with radiotherapy serving as a critical adjunctive treatment (1, 2). However, due to the anatomical proximity of the breast to the heart, radiation exposure during treatment is strongly associated with an increased risk of radiation-induced heart disease, particularly in left-sided breast cancer (3). Modern radiotherapy planning systems, utilizing inverse intensity-modulated radiotherapy (IMRT) and dose optimization algorithms, effectively limit radiation exposure to organs at risk (OARs) while ensuring adequate target dose coverage, and have become a standard in clinical practice (4–6). To further reduce cardiac dose, the deep inspiration breath-hold (DIBH) technique has emerged as an essential approach in recent breast cancer radiotherapy (7, 8). DIBH increases lung volume, expanding the distance between the target and the heart, which enhances dose attenuation in the target area and minimizes radiation exposure to the heart (9, 10). The combination of DIBH with IMRT dose optimization algorithms has been shown to significantly reduce radiation to the heart and its substructures, thereby lowering the risk of radiation-induced heart disease (11).

The Monaco treatment planning system (TPS), which uses the Monte Carlo dose calculation algorithm, is one of the most widely employed systems in clinical practice, providing highly accurate dose optimization results that closely reflect actual radiation-induced damage (12). The system’s Serial function is a key biological optimization tool, especially for dose constraints applied to the heart and its substructures. In Monaco-Serial, a K value of 1 is commonly used for setting average dose constraints to the heart and its substructures in left breast cancer free-breathing IMRT (FB-IMRT) (13, 14). However, with DIBH-IMRT, significant anatomical changes, such as the increased distance between the heart and the target, can render previous settings suboptimal, and to date, there is a lack of studies addressing this issue.

This study aims to identify the optimal K value setting for Monaco-Serial in DIBH-IMRT for left-sided breast cancer by retrospectively analyzing 41 patients who underwent DIBH-IMRT. Radiotherapy plans were designed using different Monaco-Serial K values, and dosimetric comparisons were made. Furthermore, the correlation between changes in dose-volume parameters of OARs and anatomical variations post-DIBH was explored. The goal is to provide data-driven insights for optimizing Monaco-Serial settings in left-sided breast cancer DIBH-IMRT, thus supporting the clinical application of the Monaco system in designing DIBH-IMRT treatment plans.

## 2 Materials and methods

**Figure 1:**
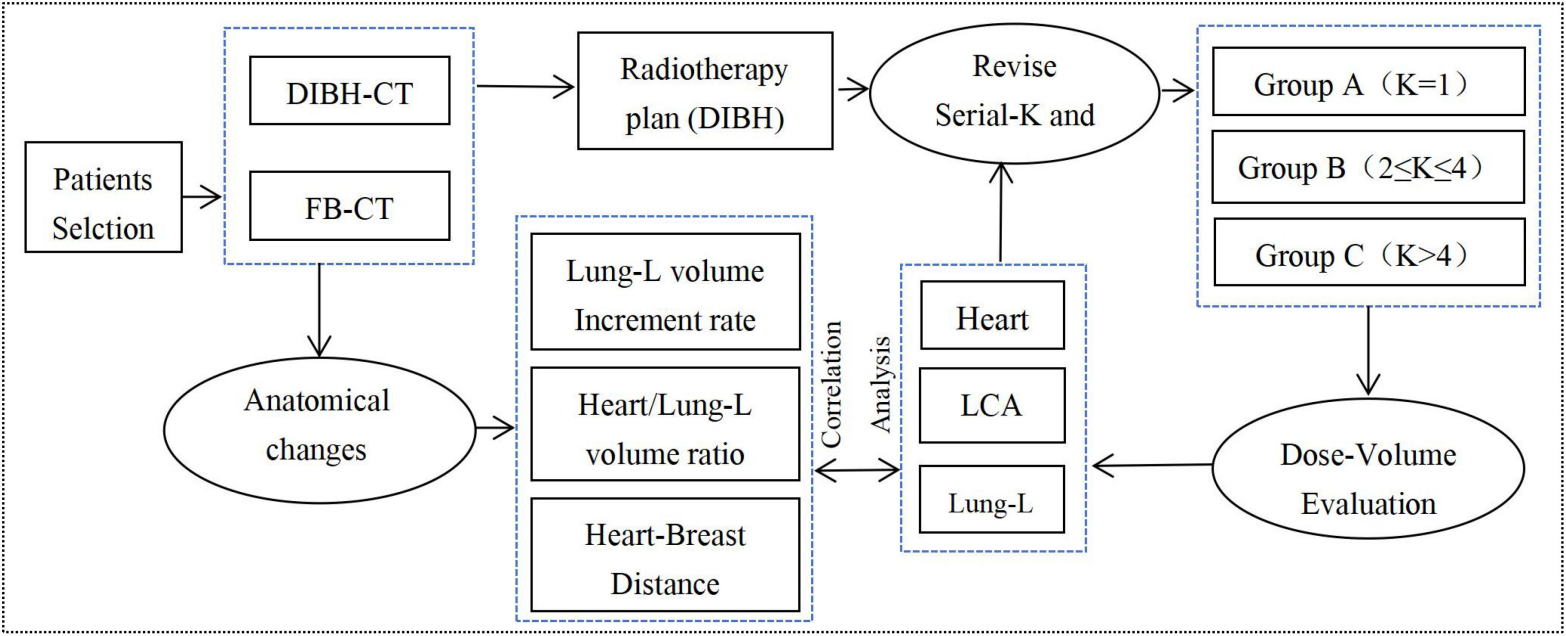
Flow chart of the radiotherapy treatment planning.

This study was approved by the Ethics Committee of the researchers’ hospital (Approval No. 2024-145K) and prospectively registered at ClinicalTrials (identifier NO.NCT06796257), adhering to ICMJE guidelines. It included 41 patients with left-sided breast cancer who underwent breast-conserving surgery followed by whole-breast radiotherapy between August 2022 and December 2024, with a mean age of 43.3 years (range: 29–72 years). All participants demonstrated good compliance and successfully completed the entire DIBH treatment protocol. The study workflow is outlined in **Figure 1**, which encompasses CT simulation and positioning under both free-breathing (FB) and DIBH conditions, radiotherapy plan design and evaluation, as well as the analysis of anatomical changes and dose reduction rates following DIBH.

### 2.1 CT simulation and target delineation

All patients were positioned and immobilized using a vacuum bag combined with a single-board system (R612, Klarity, China). CT simulation images were acquired with a CT scanner (Somatom, Siemens, Germany), covering the scan range from the inferior margin of the mandible to the inferior margin of the liver. Immediately after completing the CT scan in the FB position, each patient underwent a chest-breathing DIBH CT scan. Prior to the DIBH scan, patients received breathing training and were required to achieve breath-holding for more than 20 seconds in three consecutive attempts. Those who met this criterion were deemed eligible for DIBH CT scanning. Breathing gating during DIBH was performed using a laser-based surface scanner (Sentinel, C-RAD AB, Sweden), with the gating point monitored at the mid-sternum. The CT scan was conducted at a voltage of 110 kV, with a slice thickness of 3 mm. For the DIBH scan, an iodinated contrast agent was administered to enhance image quality. Both DIBH-CT and FB-CT images were subsequently transferred to the radiotherapy planning system (Monaco 6.0, Elekta, Sweden).

On the DIBH-CT images, radiation oncologists reviewed and contoured the OARs and target volumes. The Gross Tumor Volume (GTV), Clinical Target Volume (CTV) and OARs were defined according to Radiation Therapy Oncology Group (RTOG) standards (15), while the Planning Target Volume (PTV) and Planning Gross Tumor Volume (PGTV) were generated by expanding the CTV and GTV by 10 mm and contracting the subcutaneous volume by 5 mm. The heart, left lung, right lung, and body contour were auto-contoured using an automatic segmentation tool (AccContour, China), with manual adjustments made by the radiation oncologist. The left anterior descending artery (LAD) was manually contoured by the radiation oncologist. All OARs and target delineations were reviewed and approved by senior radiation oncology experts.

### 2.2 Radiotherapy plan design

All patients received a prescribed dose of 5000 cGy in 25 fractions for the PTV. Postoperative breast-conserving surgery patients received an additional boost to the PGTV, totaling 5750 cGy in 25 fractions. Target volume coverage adhered to ICRU recommendations, ensuring that the maximum dose did not exceed 107%, and the 95% isodose line covered the PTV/PGTV. The DIBH radiotherapy plans for all patients were designed by the same radiation therapy physicist using 6MV-FFF photon energy with 6 tangential IMRT fields based on the DMLC technique. All dose calculations were performed using the Monte Carlo algorithm on Monaco 6.0, with a 3 mm calculation grid and a statistical uncertainty of 1%.

During radiotherapy planning, the dose limits for OARs followed the guidelines for breast cancer radiotherapy (Chinese Medical Association, 2020 edition) (16). The dose-volume objectives, optimization functions, and parameter settings are summarized in **Table 1**. Three different radiotherapy plans were designed for each patient based on varying Monaco-Serial K values for cardiac dose constraints: Group A (K=1), Group B (2≤K ≤ 4), and Group C (K>4). For heart dose constraints, the Equivalent Uniform Dose (EUD) was adjusted according to dose optimization principles, with modifications made in response to changes in K values. For the lung (Lung-L), LAD, and spinal cord dose constraints, EUD, MOD (Mean Organ Damage), and Maximum Dose values were fine-tuned to minimize OARs dose-volume while maintaining adherence to dose optimization principles, as outlined in **Table 1**. All three radiotherapy plans were reviewed and approved by the same senior radiation oncologist before undergoing dosimetric analysis. Additionally, the plans were independently verified using the 3D dose verification system (Evolution, IBA, Germany), ensuring consistency and accuracy. The verification criterion required a gamma passing rate (2mm/3%) of over 95%.

**Table 1:** Dose-volume constraints for OARs and the optimization functions and parameters used in this study.

Target Volume	Dose-Volume Target	Constraint Functions and Parameter Settings
Lung-L	Dmean ≤ 1500 cGy (1200 cGy), V3000 cGy ≤ 20% (15%), V2000 cGy ≤ 30% (25%), V500 cGy ≤ 50% (50%)	Parallel: ①EUD=2800 cGy/MOD=16%; K=3 ②EUD=1800 cGy/MOD=25%; K=3 ③EUD=480 cGy/MOD=48%; K=3 Serial: EUD=1000~1400 cGy; K=1
Lung-R	Dmean ≤ 500 cGy (300 cGy)	Serial: EUD=500 cGy; K=1
Heart	Dmean ≤ 800 cGy (400 cGy)	Serial: EUD=300~1000 cGy; Group A: K=1; Group B: 2≤K ≤ 4; Group C: K>4
LAD	Dmean ≤ 2500 cGy (1000 cGy)	Serial: EUD=400~1000 cGy; K=1
Spinal cord	Dmax ≤ 4000 cGy(3000 cGy)	Maximum Dose: 3000 cGy

In the “Dose-Volume Target” column, values outside the parentheses are the guideline-recommended values, and values inside the parentheses represent the experience values from our center for left breast DIBH radiotherapy plans. EUD, Equivalent Uniform Dose; MOD, Mean Organ Damage; K, Value of Power Law Exponent.

### 2.3 Measurement and calculation of anatomical structure changes

**Figure 2:**
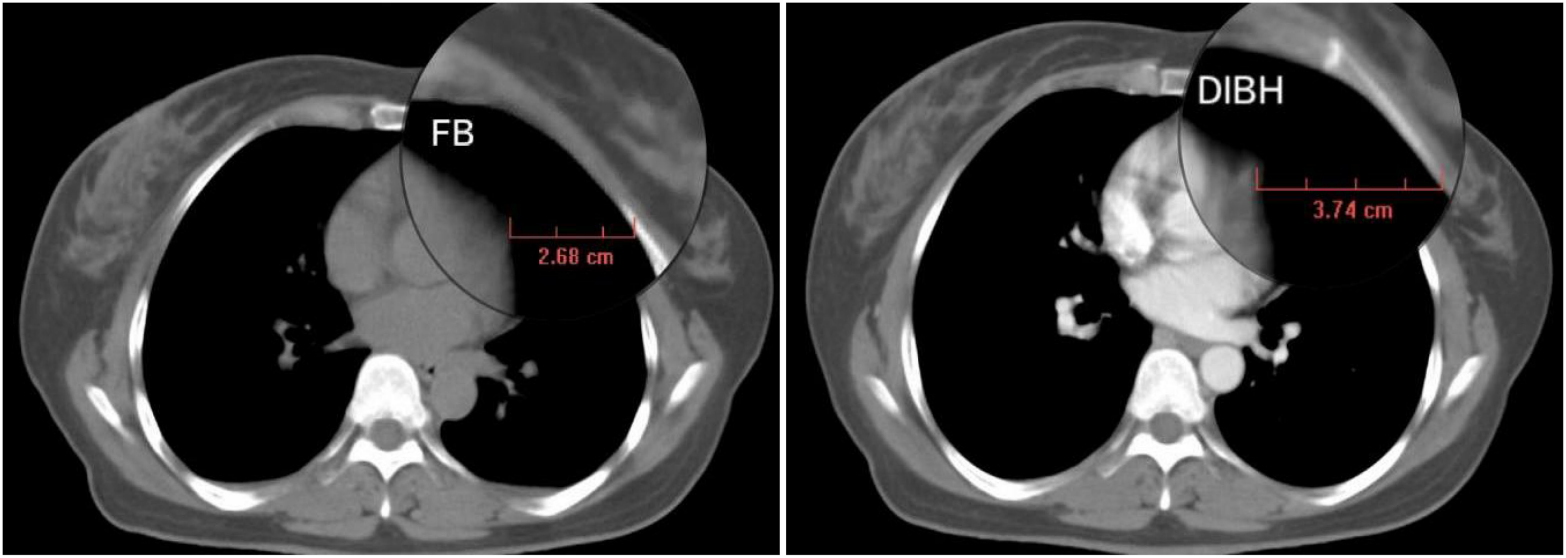
Example of the measurement method for heart-breast distance under FB and DIBH conditions.

The following three anatomical change parameters after DIBH were measured and calculated for all patients using CT images from both FB and DIBH:

(1) Lung-L volume increment rate = (DIBH Lung-L volume - FB Lung-L volume)/FB Lung-L volume; (2) Heart/Lung-L volume ratio = DIBH Heart volume/DIBH Lung-L volume; (3) Heart-breast distance increment = DIBH Heart-breast distance - FB Heart-breast distance. The measurement method for the Heart-breast distance in both FB and DIBH is illustrated in **Figure 2**, which is provided in the supplementary materials. The CT slice used for this measurement is the transverse cross-section at the midline of the breast in the head-foot direction.

### 2.4 Dosimetric and correlation analysis

The dosimetric differences in three OARs—Heart, LAD, and Lung-L—were statistically analyzed and compared across the radiotherapy plans of Groups A, B and C. Group A served as the reference, and the dose-volume reduction rates for the three OARs in Groups B and C were calculated after adjusting the K value. Additionally, the dose-volume reduction rates for OARs in Groups B and C were correlated with DIBH-related anatomical changes: Lung-L volume increment rate, Heart/Lung-L volume ratio, and Heart-breast distance increment.

### 2.5 Statistical analysis

Data were first tested for non-normal distribution using the Shapiro-Wilk test. The Mann-Whitney U test was applied to analyze differences between two groups, while the Kruskal-Wallis H test was used for comparisons among multiple groups. When significant differences were detected among groups, pairwise comparisons were performed with Bonferroni correction to adjust for multiple testing. Pearson correlation analysis was performed to investigate the relationship between dose-volume reduction rates of OARs and anatomical indicators. All statistical analyses were conducted using SPSS 27.0, with a p-value of < 0.05 considered statistically significant unless otherwise specified after correction.

## 3 Results

### 3.1 Dosimetric statistics for All DIBH-IMRT treatment plans

#### 3.1.1 Dose-volume results of OARs in the three radiotherapy plans

**Figure 3:**
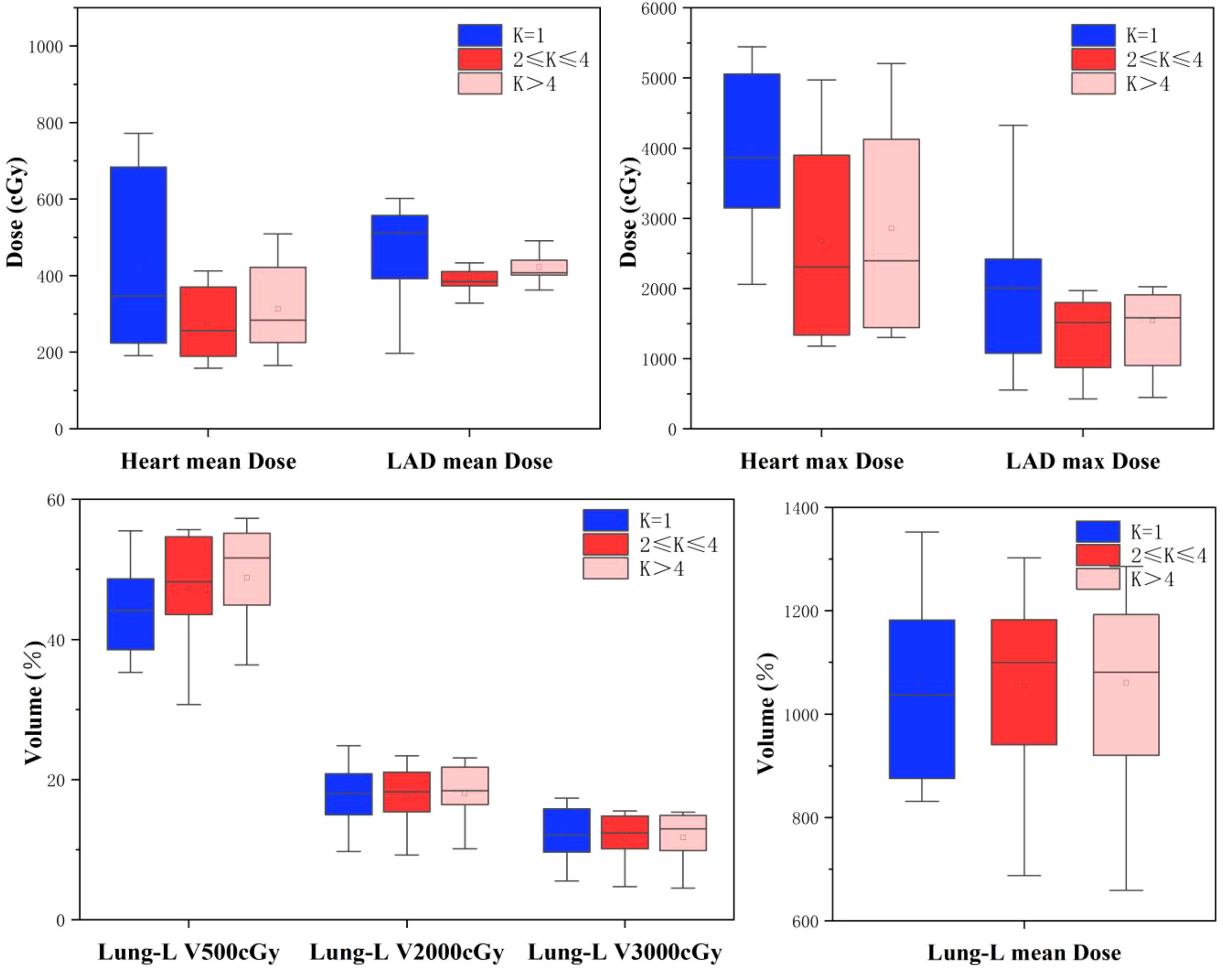
Boxplots of treatment planning data for OARs, for the 41 left breast cancer patients included in this study, with DIBH, different serial-K value ranges and 6 fields tlMRT.

**Table 2** and **Figure 3** summarize the dose-volume data for the heart, LAD, and ipsilateral lung under the three treatment plans: Group A, Group B, and Group C. Compared to Group A, both Group B and Group C demonstrated reductions in the average and maximum doses to the Heart and LAD, with Group B showing better dose control than Group C. In Group B, the average dose to the Heart and LAD decreased to 273.9 ± 91.0 cGy and 411.3 ± 127.8 cGy (*p* < 0.05), respectively, while the maximum dose decreased to 2676.2 ± 1380.7 cGy and 1483.3 ± 736.3 cGy (*p* < 0.05), respectively.

**Table 2:** Summary of treatment planning data for OARs, for the 41 left breast cancer patients included in this study, with DIBH, different Serial-K value ranges and 6 fields tlMRT.

Dose-Volume for OARs	Group A (K=1)	Group B (2≤K ≤ 4)	Group C (K>4)	*p*
Heart mean Dose(cGy)	423.4 ± 213	**273.9 ± 91.0**	312.7 ± 110.2	<0.05
Heart max Dose(cGy)	3980.0 ± 1038.0	**2676.2 ± 1380.7**	2856.1 ± 1440.9	<0.05
LAD mean Dose(cGy)	530.3 ± 197.6	**411.3 ± 127.8**	440.6 ± 134.0	<0.05
LAD max Dose(cGy)	2012.9 ± 1008.0	**1483.3 ± 736.3**	1537.3 ± 755.6	<0.05
Lung-L mean Dose(cGy)	1033.1 ± 162.8	1042.0 ± 165.4	1126.9 ± 183.0	=0.09
Lung-L V500cGy(%)	**44.2 ± 6.2**	46.7 ± 7.2	49.7 ± 8.2	<0.05
Lung-L V2000cGy(%)	17.5 ± 4.1	17.5 ± 4.3	18.7 ± 4.4	=0.32
Lung-L V3000cGy(%)	12.1 ± 3.6	11.4 ± 3.6	12.7 ± 4.0	=0.56

The dose-volume parameters for OARs are presented as mean values ± one standard deviation. Most favourable value was marked in bold.

For the ipsilateral lung (Lung-L), the V500 cGy in Group A was 44.2 ± 6.2% (*p* < 0.05), with an average dose of 1033.1 ± 162.8 cGy, which was the lowest among the three groups. In Group B, although the V500 cGy and average dose for Lung-L showed a slight increase, the values remained within an acceptable range.

In summary, adjusting the Serial-K value for the Heart to the Group B range (2 ≤ K ≤ 4) significantly optimized the dose constraints for the heart and its substructures, while only causing a minimal increase in the dose-volume to the ipsilateral lung.

#### 3.1.2 Dose-volume reduction rates of major OARs after adjusting the cardiac serial-K value

**Figure 4:**
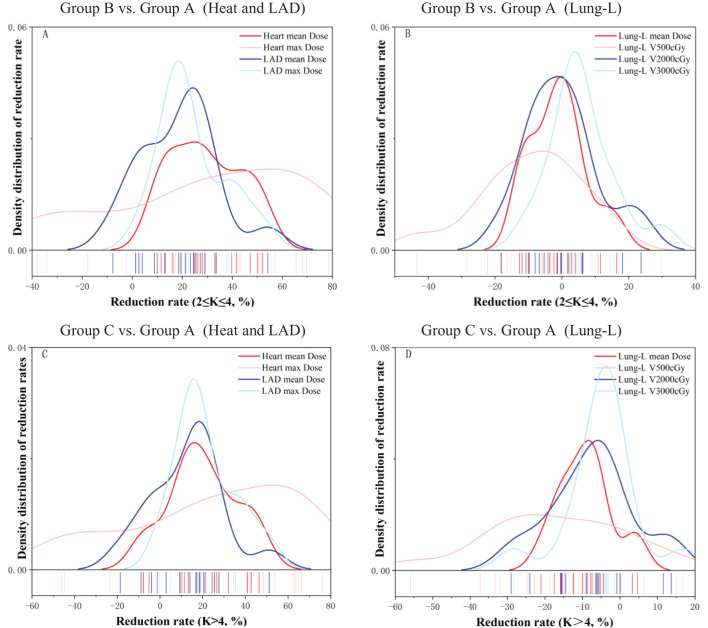
This figure displays the density distribution curves of dose-volume reduction rates for OARs after optimization of radiotherapy plans using different serial-K values (divided into two Groups: B、C), relative to Group A.

The dose-volume reduction rate was calculated using the following formula: Dose-volume Reduction Rate = ((Adjusted Dose - Original Dose)/Original Dose) * 100%. **Table 3** summarizes the dose-volume reduction rates for the three OARs—Heart, LAD, and Lung-L—in Group B and Group C compared to Group A. **Figure 4** displays the density distribution curves of the dose-volume reduction rates for OARs in Group B and Group C relative to Group A. As shown in **Table 3**, compared to Group A, Group B demonstrated an average reduction rate of 29.4% ± 14.2% for the Heart and 18.7% ± 15.5% for the LAD, with maximum dose reduction rates of 29.3% ± 38.0% and 24.0% ± 13.4%, respectively. For Lung-L, the average reduction rate for V500 cGy in Group B compared to Group A was -8.6 ± 15.3% and -15.6 ± 17.5%, respectively. **Figure 4** further illustrates that Group B significantly outperformed Group C in terms of dose reduction rates for both the Heart and LAD.

**Table 3:** Summary of reduction rates in dose-volume of OARs after treatment plan optimization by varying Serial-K values (two groups: B、C), relative to treatment plan optimization with Group A, for the 41 patients with left breast cancer included in this study, with DIBH and 6 tlIMRT fields.

Dose-Volume for OARs	Group B (2≤K ≤ 4, %)	Group C (K>4, %)	*p*
Heart mean Dose(cGy)	**29.4 ± 14.2**	20.1 ± 16.0	<0.05
Heart max Dose(cGy)	**29.3 ± 38.0**	24.5 ± 40.1	<0.05
LAD mean Dose(cGy)	**18.7 ± 15.5**	12.7 ± 17.0	<0.05
LAD max Dose(cGy)	**24.0 ± 13.4**	21.1 ± 14.1	<0.05
Lung-L mean Dose(cGy)	-1.1 ± 8.3	-8.7 ± 7.1	<0.05
Lung-L V500cGy(%)	-8.6 ± 15.3	-15.6 ± 17.5	<0.05
Lung-L V2000cGy(%)	0.1 ± 11.0	-7.4 ± 11.5	<0.05
Lung-L V3000cGy(%)	5.9 ± 9.8	-4.8 ± 9.5	<0.05

Reduction rates in dose-volume of OARs are shown as mean values with one standard deviation for OARs. Most favourable value was marked in bold.

In conclusion, these results underscore that adjusting the Serial-K value for the Heart to the Group B range (2 ≤ K ≤ 4) not only optimized dose constraints for the heart and its substructures, but also led to only a slight increase in Lung-L V500 cGy.

### 3.2 Anatomical parameter measurements and calculation results after DIBH

The study results demonstrated significant anatomical changes following DIBH compared to the FB state in 41 patients. The Lung-L volume increment rate was 81.7 ± 36.2%, the Heart/Lung-L volume ratio was 0.03 ± 0.09, and the Heart-breast distance increment was 1.0 ± 0.5 cm. These measurements indicate significant anatomical changes after DIBH compared to the FB state.

### 3.3 Correlation analysis results between anatomical and dosimetric parameters

**Figure 5:**
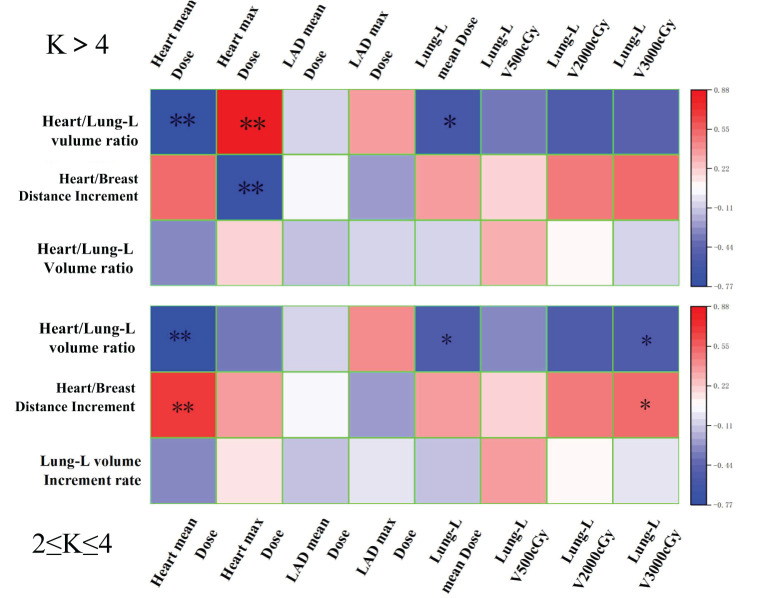
The correlation heatmap shows the pearson’s correlation between the reduction rates in dose-volume of OARs (compared to Group A), and the modifications in anatomical structures (after DIBH) from radiotherapy planning for different Serial-K values (Group B and C). ** Significant correlation at the 0.01 level. * Significant correlation at the 0.05 level.

**Figure 5** shows the correlation between the dose-volume reduction rates of the Heart, LAD, and Lung-L in Group B (2≤K ≤ 4) and Group C (K>4) compared to Group A (K=1), after adjusting the Monaco-Serial K value for constraining the heart, and the three anatomical parameters: Heart/Lung-L volume ratio, Heart/breast Distance Increment, and Lung-L volume Increment rate.

As shown in **Figure 5**, in Group B (2≤K ≤ 4), the Heart/breast Distance is strongly positively correlated with both the average and maximum heart dose reduction rates. The Heart/Lung-L volume ratio is strongly negatively correlated with these dose reduction rates. After DIBH, the changes in these two anatomical parameters enabled Group B (2≤K ≤ 4) to effectively constrain both the average and maximum heart doses, with a more significant effect observed on the average dose, which aligns with the expected effects of anatomical variations induced by the DIBH technique. In contrast, Group C (K>4) did not achieve optimal constraints for either the average or maximum doses.

Therefore, the correlation analysis indicates that adjusting the Monaco-Serial K value for heart constraint to the Group B (2≤K ≤ 4) range results in the best correlation between anatomical parameters and heart and substructure doses. This further confirms that Group B (2≤K ≤ 4) is the optimal range for the Monaco-Serial K value in DIBH radiotherapy plans for left-sided breast cancer.

## 4 Discussion

In breast cancer radiotherapy, the risk of cardiac injury is primarily linked to the mean dose to the heart, which increases linearly with radiation dose. Studies have demonstrated that for every 100 cGy increase in the mean radiation dose to the heart, the likelihood of adverse coronary events rises by 7.4%, with the risk potentially persisting for decades (3). Since radiotherapy implementation is based on treatment plan design, radiation-induced heart damage is closely associated with the optimized dose distribution in radiotherapy plans (17–19).

With current photon radiotherapy technology, combining DIBH with IMRT dose optimization algorithms can effectively limit radiation exposure to the heart and its substructures, potentially significantly reducing the risk of radiation-induced heart disease. In IMRT dose optimization, traditional physical optimization functions fail to fully account for the true biological response of different tissues or organs to radiation during treatment (20). However, Niemierko introduced the EUD function, which incorporates bological parameters to quantify tissue responses to radiation and optimize the dose distribution in radiotherapy plans by considering the biological characteristics of organs and the dose-response relationship. This results in a more accurate reflection of the biological effects of radiation on tissues and organs, thus improving treatment precision and safety (21, 22). Numerous studies have confirmed that the EUD function can effectively control the dose to OARs in breast cancer radiotherapy, reducing radiation-induced damage to structures like the heart (23, 24). For example, Lee et al. (23) compared the quality and performance of dose-volume (DV) plans and DV-EUD plans in breast cancer radiotherapy. Their results showed that DV-EUD plans provided better protection for OARs, reducing lung and heart doses compared to standard DV plans. Similarly, Mihailidis et al. (24) found that EUD-based plans offered superior protection for OARs while maintaining target coverage. Consequently, the use of EUD-based biological optimization functions has become widely accepted and applied in clinical research (25–29).

In this study, we employed the Monaco TPS, which supports advanced biological optimization models such as the Lyman-Kutcher-Burman (LKB) model. Among these, the Monaco-Serial function is a biological optimization model for serial organs based on the concept of EUD. A key parameter in this model is K—typically ranging from 1 to 20—which modulates the organ’s sensitivity to different dose distributions. A lower K value indicates greater sensitivity to the mean dose. Previous studies have successfully applied a K value of 1 in free-breathing IMRT plans for left-sided breast cancer to achieve effective optimization of heart dose-volume constraints. However, the optimal K value for DIBH-IMRT planning remains uncertain and warrants further investigation (30–32). In this study, when applying Monaco-Serial to optimize the heart dose in left-sided breast cancer DIBH-IMRT, adjusting the Serial-K from K=1 to the range of 2≤K ≤ 4 (Group B) achieved the best dose constraints for the heart and its substructures, with only a slight increase in Lung-L V500 cGy (still <50%). This finding differs from previous studies and experiences with FB-IMRT. Research by Tanguturi et al. (33) noted that changes in lung volume between FB and DIBH significantly affect heart dose optimization. Cao et al. (34) found that the increased distance between the heart and the chest wall in DIBH significantly impacted heart dose optimization. Thus, the improved heart dose constraints observed in our study, with Serial-K adjusted to 2≤K ≤ 4, may be attributed to anatomical changes induced by DIBH compared to FB.

To further explore the impact of anatomical changes after DIBH on the optimal K value, we analyzed the correlation between anatomical structure changes and the dose-volume reduction rates of OARs with increased K values. The study found that after DIBH, as the Heart/Lung-L volume ratio and Heart/breast Distance Increment increased, a modest increase in K (within the 2≤K ≤ 4 range) effectively constrained both the mean and maximum heart doses, with the most significant effect on mean dose constraints. These results align with previous studies and clinical expectations (22, 23). However, for Group C (K>4), further increases in K led to stronger constraints on the maximum dose, which impacted the dose distribution of the target area. This caused a shift in the balance between the heart dose constraint function and the target optimization function, ultimately limiting the optimization effect on the heart and its substructures. This further supports the conclusion that the optimal K value range for left-sided breast cancer DIBH-IMRT is 2≤K ≤ 4.

## 5 Conclusion

This study is the first to report the application of the Monaco-Serial biological optimization function in left-sided breast cancer DIBH-IMRT radiotherapy plans. The results demonstrate that adjusting the Serial-K value to the range of 2≤K ≤ 4 enables more effective constraints on both the mean and maximum heart doses while maintaining target dose coverage, significantly reducing the risk of radiation-induced heart damage. This finding provides valuable data to support clinical radiotherapy plan design. However, this study only focused on integer values of K. Future research will further investigate the impact of fractional K values (e.g., K=1.1, 1.2, 2.1, 2.2) on plan quality to optimize the application of the Monaco-Serial biological function. Additionally, the results may be influenced by factors such as the dose calculation algorithm, grid resolution, and dose smoothing techniques in the Monaco system, and future studies should further explore the impact of these technical parameters.

## Data Availability

The raw data supporting the conclusions of this article will be made available by the authors, without undue reservation.
